# Effects of Smartphone-Based Interventions on Physical Activity in Children and Adolescents: Systematic Review and Meta-analysis

**DOI:** 10.2196/22601

**Published:** 2021-02-01

**Authors:** Zihao He, Hua Wu, Fengyu Yu, Jinmei Fu, Shunli Sun, Ting Huang, Runze Wang, Delong Chen, Guanggao Zhao, Minghui Quan

**Affiliations:** 1 School of Physical Education NanChang University NanChang China; 2 Rehabilitation Medicine Center The Second Affiliated Hospital of Jiaxing University Jiaxing China; 3 JiangXi Institute of Sport Science NanChang China; 4 School of Kinesiology Shanghai University of Sport Shanghai China

**Keywords:** adolescents, children, mHealth, physical activity, smartphone

## Abstract

**Background:**

About 70% of children and adolescents worldwide do not meet the recommended level of physical activity (PA), which is closely associated with physical, psychological, and cognitive well-being. Nowadays, the use of technologies to change PA is of interest due to the need for novel, more effective intervention approaches. The previous meta-analyses have examined smartphone-based interventions and their impact on PA in adults, but evidence in children and adolescents still needs further research.

**Objective:**

This systematic review and meta-analysis aimed to determine the effectiveness of smartphone-based interventions for improving PA in children and adolescents.

**Methods:**

Five electronic databases (PubMed, Web of Science, OVID, Scopus, and the China National Knowledge Infrastructure) were searched up to June 29, 2020. Randomized controlled trials with a control group that examine the effect of smartphone interventions on PA among children and adolescents were included. Bias risks were assessed using the Cochrane collaboration tool. Meta-analysis was performed to assess the pooled effect on PA using a random effects model. Subgroup analyses were conducted to examine the potential modifying effects of different factors (eg, types of intervention, intervention duration, age, measurement, study quality).

**Results:**

A total of 9 studies were included in this review, including 4 mobile app interventions, 3 SMS text messaging interventions, and 2 app + SMS text messaging interventions. In general, the risk of bias of included studies was low. Compared with the control group, the use of smartphone intervention significantly improved PA (standardized mean difference [SMD] 0.44, 95% CI 0.11-0.77, *P*=.009), especially for total PA (TPA; weighted mean difference [WMD] 32.35, 95% CI 10.36-54.33, *P*=.004) and daily steps (WMD 1185, 95% CI 303-2068, *P*=.008), but not for moderate-to-vigorous PA (WMD 3.91, 95% CI –1.99 to 9.81, *P*=.19). High statistical heterogeneity was detected (*I*^2^=73.9%, *P*<.001) for PA. Meta-regression showed that duration (β=–.08, 95% CI –0.15 to –0.01, n=16) was a potential factor for high heterogeneity. The results of subgroup analyses indicated that app intervention (SMD 0.76, 95% CI 0.23-1.30, *P*=.005), children (SMD 0.64, 95% CI 0.10-1.18, *P*=.02), “≤8 weeks” (SMD 0.76, 95% CI 0.23-1.30, *P*=.005), objective measurement (SMD 0.50, 95% CI 0.09-0.91, *P*=.02), and low risk of bias (SMD 0.96, 95% CI 0.38-1.54, *P*=.001) can significantly improve PA.

**Conclusions:**

The evidence of meta-analysis shows that smartphone-based intervention may be a promising strategy to increase TPA and steps in children and adolescents. Currently, app intervention may be a more effective strategy among smartphone intervention technologies. To extend the promise of smartphone intervention, the future needs to design comparative trials among different smartphone technologies.

**Trial Registration:**

PROSPERO CRD42019148261; https://tinyurl.com/y5modsrd

## Introduction

Childhood and adolescence are critical periods of growth. Engaging in enough physical activity (PA) has been demonstrated to benefit children’s physical and mental health, such as reducing health risks, preventing obesity, and developing cognitive function [1,2]. To achieve health benefits through PA, the World Health Organization (WHO) recommends that children and adolescents accumulate moderate-to-vigorous intensity PA (MVPA) exceeding 60 minutes per day [3]. However, the rising prevalence of physical inactivity is a serious concern worldwide. Globally, about 70% of children and adolescents do not meet the recommendations on PA [4]. For example, a Chinese PA and fitness survey showed that two-thirds of children and adolescents did not meet the recommended PA [5]. Insufficient PA is closely related to obesity, coronary heart disease, and other health problems [6-8]. Hence, it is of paramount importance to promote and facilitate PA safely and effectively during this critical period. In response to this difficult situation, researchers have carried out a series of intervention studies on PA. However, many intervention strategies not only suffer from high cost, but are also difficult to maintain and implement on a large scale [9-11]. Therefore, how to use cost-effective and innovative intervention strategies to improve the PA level of children and adolescents effectively remains a major public health problem.

To date, the popularity of smartphones in the world is extremely high: 73.1% of children and adolescents own a smartphone in China [12], and this trend can also be seen in the United States [13] and other countries [14]. Given the global scale of noncommunicable diseases, there is a need to provide preventative interventions to reach a large population at a low cost. Therefore, many researchers have applied smartphone technologies, such as mobile apps and SMS text messaging, to health-related fields and have achieved rich research results, such as weight management, cancer nursing, and chronic obstructive pulmonary disease self-monitoring [15-17]. It is gratifying that more researchers have tried to introduce smartphone technology into the field of PA. The participants included not only adults [18-21], but also children and adolescents who urgently need attention [22-30]. This undoubtedly provides a new perspective for solving the aforementioned problems. Therefore, at the 65th Annual Meeting of American College of Sports Medicine (ACSM) and the 9th World Congress on Exercise is Medicine held in the United States in 2018, the promotion of smartphones for PA was highlighted [31].

To date, many researchers have explored the effect of smartphone interventions on improving the PA of children and adolescents through randomized controlled trials (RCT), but there are controversies about inconsistent research results. Some studies have found that smartphone interventions can significantly improve the level of PA relative to their baseline than the control group, such as Garde et al [22] (1758 steps/day, 95% CI 133-3384; 31.3 total PA [TPA] minutes/day, 95% CI 3.9 to 58.9), Chen et al [23] (0.4 PA day per week, 95% CI 0.15-0.66), Garde et al [25] (2934 steps/day, 95% CI 1434-4434; 46 TPA minutes/day, 95% CI 20-72), but other studies have not found a significant positive effect, such as Mendoza et al [24] (MVPA, –4.5 minutes/day, 95% CI –35.9 to 27), Direito et al [26] (MVPA, –1.82 minutes/day, 95% CI –16 to 12.36), Armstrong et al [28] (MVPA, 10 minutes/day, 95% CI –2.5 to 30), Thompson et al [29] (MVPA, 1.73 minutes/day, 95% CI –5.1 to 8.5; step, 318 steps/day, 95% CI –466 to 1102), and Newton et al [30] (step, –22 steps/day, 95% CI –1407 to 1364). Although there was 1 meta-analysis of smartphone intervention on adolescents to improve PA [32] and found a significant improvement on MVPA (standardized mean difference [SMD] 0.341, 95% CI 0.02-0.66), only 5 studies were included. Also, 2 of the 5 studies were multicomponent interventions (including smartphone and other components), which made it difficult to identify the true smartphone effect. Furthermore, this review missed some studies in the database [25,27,30]. Given the fact that there have been many new RCTs in recent years [22,24,28], and the previous reviews include comprehensive intervention strategies, it is unclear whether intervention effects were truly due to the smartphone itself, or rather the other intervention components [18]. Therefore, conducting a new meta-analysis on this topic is necessary.

The objective of this review is to evaluate the effectiveness of smartphone interventions to promote PA in children and adolescents, by using systematic review and meta-analysis to combine the most comprehensive and up-to-date literature. The findings of this study are expected to provide insights and practice for the development of future smartphone interventions.

## Methods

### Registration and Approval

This research program has been registered on the PROSPERO System Evaluation Registration Platform, registration number: CRD42019148261. This study has been reported according to the Preferred Reporting Items for Systematic Reviews and Meta-analyses (PRISMA) guidelines [33].

### Search Strategy

A systematic literature search was conducted to find out relevant studies in 5 electronic databases: PubMed, Web of Science, OVID, Scopus, and the China National Knowledge Infrastructure. The core keywords identified include children, adolescents, smartphone, and “physical activity.” PubMed MeSH database and other search engines were used to find synonyms of keywords, including the following 4 groups: (1) population: “high school” or youth or teen or “middle school” or “secondary school” or elementary or pupil or “primary school” or pediatric or preschool or kindergarten; (2) intervention: cellphone or “cellular phone” or “mobile phone” or “mobile technology” or mHealth or tablet or accelerometer or actigraphy or “activity tracker” or pedometer or “mobile application” or app or “mobile exergame” or “mobile game” or “text messaging” or “short message service” or SMS or “social media” or Facebook or WeChat; (3) outcomes: PA or activity or inactivity or exercise or sport or steps or “health behavior”; (4) study design: “randomized controlled trial.” The search period was all-inclusive up to June 29, 2020 (Multimedia Appendix 1).

Initially, 2 reviewers (ZH and GZ) searched in the databases and exported all studies to reference management software and deleted duplicate studies. Moreover, 2 independent reviewers (TH and ZH) screened the titles and abstracts identified in the electronic databases to obtain eligible articles for the full-text analysis. In addition, both reviewers manually reviewed reference lists from relevant original research and review studies. Disagreements were resolved by group discussion with a third reviewer (MQ).

### Selection Criteria of Studies

#### Inclusion Criteria

Inclusion criteria, according to PICOS (*p*opulation, *i*ntervention, *c*omparison, *o*utcomes, and *s*tudy) [34], were as follows:

1. Participants: children and adolescents aged 6-18 years, based on the PubMed MeSH definition of children (6-12 years) and adolescents (13-18 years).
2. Interventions: smartphone as the intervention tool, which used either app or SMS text messaging or both to promote PA.
3. Control groups included participants not using smartphone technology.
4. Outcomes: PA including daily steps or any intensities of PA. To be included in the meta-analysis, the outcome should be reported as steps, minutes, or hours. Studies that reported PA in other forms (eg, counts per minute, days per week) were included only in the systematic review.
5. The study design was RCTs.

#### Exclusion Criteria

1. Studies where the intervention technologies were not smartphone based (computer) or incorporated other components (eg, physical education, school seminar).
2. Studies did not report data on PA level (eg, PA score, self-efficacy on PA).
3. Studies were not written in English or Chinese.

### Data Extraction

Two authors (GZ and ZH) extracted information and data independently, including study characteristics (the first author, publication year, country, study design, contents of intervention, study duration), subject characteristics (age, sex, sample size), and outcome (measurement strategy, statistical analysis, results). Disagreement was resolved through discussion until a consensus decision was reached. In the case of missing data, this information was requested from the authors a minimum of 3 times over 4 weeks.

### Risk of Bias and Quality Assessment

The Cochrane Collaboration risk of bias tool was used to categorize the risk of bias in six domains [35]: (1) sequence generation, (2) allocation sequence concealment, (3) blinding of outcome, (4) incomplete outcome data, (5) selective outcome reporting, and (6) other potential threats to validity. The item blinding of participants and personnel were excluded because it is not feasible in these types of studies [20]. In addition, the risk of bias assessment for blinding of outcome assessment was based on the method of outcome assessment (objective or subjective) [20]. Each domain was scored as low, unclear, or high risk of bias. Overall classification of low, unclear, or high risk of bias in each study was based on the combination of the domains. Figures were generated by Review Manager software (RevMan 5.3; Nordic Cochrane). Disagreement about the risk of bias assessments was resolved by consensus or consulting the third author.

### Statistical Analyses

Random-effects models were used in this study for the meta-analysis of the included studies. For studies that only presented data through graphs (eg, Boxplot), we estimated mean and SDs using the y-axis and length of the graphs [22,25,27]. For studies that reported standard errors, CI, or quartile, we converted these data to SDs [36]. We compared the changes from baseline to endpoint data between groups. The formulas for the mean and SD pre- to post-change values were as follows: Mean_change_ = Mean_post_ – Mean_pre_ and SD_change_ = SQRT [(SD_pre_^2^ + SD_post_^2^) – (2 × Corr × SD_pre_ × SD_post_)], where the correlation coefficient was set to 0.5 based on the Cochrane Collaboration Handbook guidelines [35]. SMD and 95% CI were calculated in this study because the outcomes of the included studies are measured using different methods [37].

In the following cases, specific statistical procedures were employed: (1) When there were several publications from the same project, the study with the longest follow-up was selected; if there was no intervention during the follow-up, the result of the last intervention was selected as statistical analysis data [22]. (2) If there were multiple intervention groups in the same studies, the data were considered as independent samples for analysis. Moreover, sample sizes from control groups were evenly allocated to each intervention group in the meta-analysis to avoid artificial inflation of the true sample size [26,29]. Similarly, if a study measured 2 or more PA domains (ie, TPA, MVPA, or steps), the sample size of the control group was divided by the number of domains in which the study was measured [22,25,27,29,30]. (3) Studies that reported PA in other forms (eg, counts per minute, day per week) were included only in the systematic review, but not for meta-analysis, because the data cannot be converted into minutes per day [23].

Additionally, subgroup analysis was based on the characteristics of the review, that is, outcomes (MVPA, TPA versus steps), types of intervention (app, SMS text messaging versus app + SMS text messaging), age (children versus adolescents), intervention duration (“≤8 weeks” versus “>8 weeks”), measurement (objective versus subjective), and risk of bias (low, unclear versus high). Given the consistency of variable units between the same outcome indicator among the continuous variables in TPA, MVPA, and steps, weighted mean difference (WMD) was calculated in this subgroup for statistical analysis.

The statistical heterogeneity was examined using *I*^2^ between included studies and Cochran Q-test; it was defined as very low, low, medium, and high heterogeneity when *I*^2^ values were <25%, 25% to <50%, 50% to <75%, and ≥75%, respectively [38]. Potential sources of heterogeneity were investigated using meta-regression (eg, duration, age, BMI). Egger test was adopted to detect publication bias [39]. Additionally, the “trim and fill” method was performed to estimate the impact of publication bias on the results [40]. Furthermore, to test the robustness of the results of this study, the following methods were used to conduct sensitivity analyses: 1 article was removed each time to examine whether each article had a significant influence on the overall effect (*P*<.05).

All statistical calculations were performed using the statistical software STATA 15.1 (Release 15.1 College Station, TX, USA); *P*<.05 was defined as a significant difference.

## Results

### Overview

**Figure 1:**
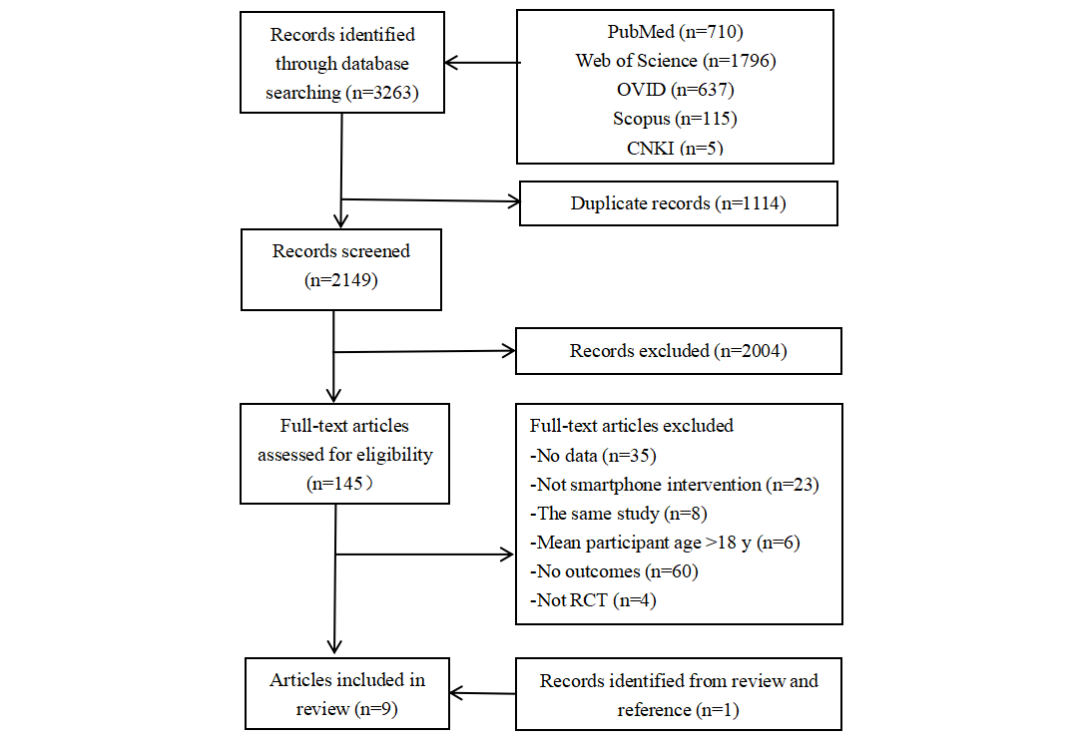
Flow chart of study selection.

There were 3263 studies produced from the electronic database search, and the titles and abstracts of 2149 of them were screened after deleting duplicates. In the screening process, a total of 2004 records were excluded, so 145 full-text studies remained to be assessed. From these, manual searches were conducted for studies that met the inclusion criteria. Finally, 9 studies were included in this review. A flow chart of the systematic literature search is displayed in Figure 1.

### Characteristics of the Included Studies

All included studies were published after 2009, 8 of which were after 2015. The study areas were distributed in 3 different countries: America (n=4) [23,24,28,29], Canada (n=3) [22,25,27], and New Zealand (n=2) [26,30]. The sample size was 558, the mean age of the participants was 13.2 years, 4 studies included children [22,25,27,28], and 5 studies included adolescents [23,24,26,29,30]. The intervention content is mainly based on smartphone technologies, app, and SMS text messaging, including 4 studies based on app [22,25-27], 3 studies based on SMS text messaging [28-30], and 2 studies based on app + SMS text messaging [23,24]. The study designs were all RCTs. The duration of interventions ranged from 2 weeks to 6 months. In addition, 1 study reporting PA days per week was not included in the meta-analysis because the data cannot be converted into minutes per day [23]. For TPA and MVPA, 6 studies objectively measured PA with an accelerometer or Tractivity activity monitor [22,24-27,29], and 3 studies used subjective assessments (questionnaires or self-reports) [23,28,30]. For the measurement of steps, 2 studies used pedometers [29,30], and 3 studies used Tractivity activity monitor [22,25,27] (Multimedia Appendix 2).

### Risk of Bias

**Figure 2:**
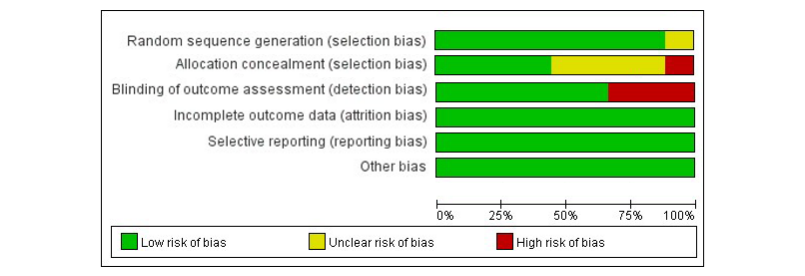
Risk of bias graph: each risk of bias item is presented as percentages.

**Figure 3:**
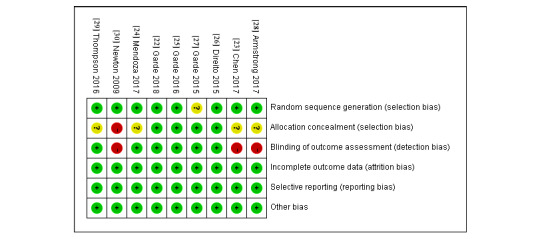
Risk of bias of included studies. Green: low risk of bias; yellow: unclear risk of bias; red: high risk of bias.

Figures 2 and 3 show the risk of bias assessment of the 9 included studies; of these, 3 studies were classified as having a low risk of bias, 4 studies were classified as having an unclear risk of bias, and 2 had a high risk of bias rating. Three studies were subjective measurement methods, so the blinded outcome assessment was rated as high risk of bias.

### Result of Meta-analysis on PA

#### The Summary Effect Analysis

**Figure 4:**
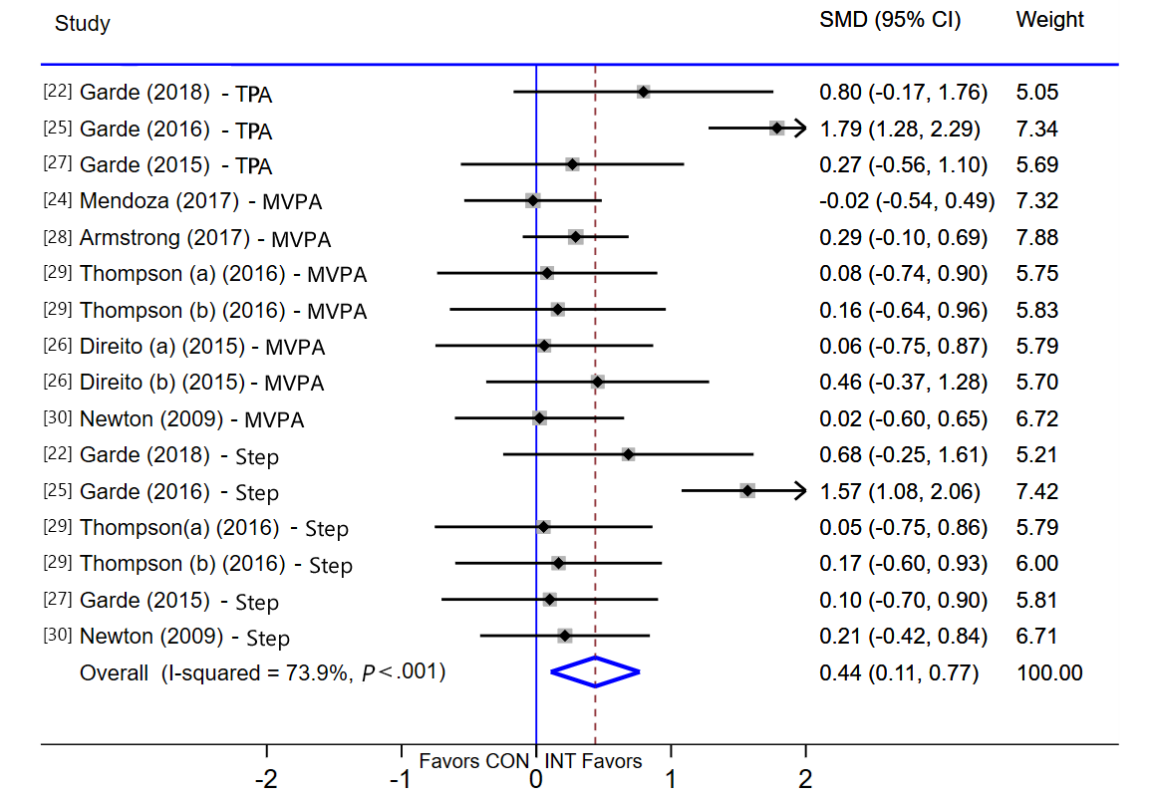
Meta-analysis of effects of intervention versus control on physical activity (PA). MVPA: moderate-to-vigorous-intensity physical activity; SMD: standardized mean difference; TPA: total physical activity.

A random-effects meta-analysis, including 8 studies (16 effects), demonstrated that there was a significant improvement in PA in the intervention group compared to the control group (SMD 0.44, 95% CI 0.11-0.77, *P*=.009), and high statistical heterogeneity was detected (*I*^2^=73.9%, *P*<.001; Figure 4). Meta-regression showed that duration (β=–.08, 95% CI –0.15 to –0.01, n=16) was a potential factor for high heterogeneity. The Egger test showed that there was no significant publication bias between the studies (*P*=.28).

#### Subgroup Analysis

The results of subgroup analysis of the effects on outcomes are shown in Table 1. Compared with the control group, subgroups of TPA (WMD 32.35, 95% CI 10.36-54.33, *P*=.004), step (WMD 1185, 95% CI 303-2068, *P*=.008), app intervention (SMD 0.76, 95% CI 0.23-1.30, *P*=.005), children (SMD 0.64, 95% CI 0.10-1.18, *P*=.002), “≤8 weeks” (SMD 0.76, 95% CI 0.23-1.30, *P*=.005), objective measurement (SMD 0.50, 95% CI 0.09-0.91, *P*=.02), and low risk of bias (SMD 0.96, 95% CI 0.38-1.54, *P*=.001) can significantly increase PA.

**Table 1:** Subgroup analyses on the effect of intervention versus control on PA in children and adolescents.

Potential modifiers		Studies, n	Effect size (95% CI)	*I*^2^ (%)	*P*-value heterogeneity
All studies		8	0.44 (0.11 to 0.77)	73.9	<.001
**Outcome^a^**					
	TPA^b^	3	32.35 (10.36 to 54.33)	61.8	.07
	MVPA^c^	7	3.91 (–1.99 to 9.81)	0.0	.94
	Step	6	1185 (303 to 2068)	43.0	.12
**Intervention**					
	App	4	0.76 (0.23 to 1.30)	76.4	<.001
	SMS text messaging	3	0.18 (–0.06 to 0.42)	0.0	.99
	App + SMS text messaging	1	–0.03 (–0.54 to 0.49)	—	—
**Age**					
	Children	3	0.64 (0.10, 1.18)	74.1	<.001
	Adolescents	5	0.32 (–0.12, 0.75)	74.8	.002
**Duration**					
	≤8 weeks	4	0.76 (0.23, 1.30)	76.4	<.001
	>8 weeks	4	0.14 (–0.07, 0.36)	0.0	.99
**Measurement**					
	Objective	6	0.50 (0.09, 0.91)	76.6	<.001
	Subjective	2	0.22 (–0.08, 0.51)	0.0	.78
**Risk of bias**					
	Low	3	0.96 (0.38, 1.54)	74.9	.001
	Unclear	3	0.09 (–0.19, 0.37)	0.0	.99
	High	2	0.22 (–0.08, 0.51)	0.0	.78

^a^Outcome: There are studies reporting 2 outcomes, so the total exceeds the total number of included studies; besides, only this subgroup was calculated using weighted mean difference (WMD), whereas for others SMD is reported.

^b^TPA: total physical activity.

^c^MVPA: moderate-vigorous intensity physical activity.

#### Robustness of the Results

Sensitivity analyses were conducted to test the robustness of the findings. One study was removed each time to perform a meta-analysis again. The results of the effect did not change significantly, which indicates that the results of the meta-analysis in this study were reliable (Multimedia Appendix 3).

## Discussion

### Principal Findings

The primary objective of this study was to determine the effectiveness of the smartphone-based intervention in improving PA in children and adolescents. The results of this study indicated that smartphone-based intervention has a significant effect on PA, especially for TPA and steps, but not for MVPA.

### Comparison With Previous Systematic Review and Meta-analysis

The findings of this study indicated that smartphone-based intervention has a positive effect on PA in children and adolescents, and our results are a valuable extension of recently published systematic reviews and meta-analysis. Previous similar studies mainly focused on the intervention effect of smartphone, app, and a combination of app and wearables on MVPA and step counts in adults, but the results of the studies were not consistent. Gal et al [20] (age range 19-79 years) reported that smartphone-based intervention was effective in promoting MVPA (SMD 0.43, 95% CI 0.03-0.82), whereas a nonsignificant difference on MVPA was observed in Romeo et al [18] (age range 22-63 years; mean difference [MD] –2.16, 95% CI –15.68 to 11.36; MD –3.16, 95% CI –7.85 to 0.63), Flores et al [19] (mean 39 years; SMD 0.40, 95% CI –0.07 to 0.87), and Direito et al’s [41] study (age range 8.4-71.7 years; SMD 0.37, 95% CI –0.03 to 0.77). Besides, Gal et al [20] (19-79 years) and Feter et al [21] (mean 40.7 [SD 14.4] years) reported that smartphone-based intervention has a significant positive effect on steps in adults (SMD 0.51, 95% CI 0.12-0.91; MD 735, 95% CI 28-1243, respectively). However, Romeo et al [18] (age range 22-63 years) and Direito et al [41] (age range 8.4-71.7 years) did not find these results (MD 477, 95% CI –230 to 1183 and SMD 0.14, 95% CI –0.01 to 0.29, respectively). The possible explanation is that the intervention effects of smartphone, app, or app plus other components are the difference [21,42]. It is necessary to conduct controlled trials between different interventions. In addition, although a significant MVPA increase was not observed in most studies, we cannot ignore the potential health-promoting effects of increased other intensity PA by smartphone interventions. Recent epidemiological evidence indicated the potential benefits of increasing light-intensity PA (LPA), including association with decreased systolic blood pressure, diastolic blood pressure, markers of lipid, and glucose metabolism [43,44]. Therefore, how to improve LPA is also the focus of future research.

At present, only Shin et al’s [32] study (10-19 years) focused on children and adolescents, and a significant improvement effect was found on MVPA (SMD 0.34, 95% CI 0.02-0.66). However, Shin et al [32] only included 5 studies, and it is difficult to identify the real smartphone intervention effect because 2 of these 5 studies were multicomponent interventions (including smartphones and other components). Hence, to fill up the research gaps from the previous meta-analysis, this study included more studies published in recent years and determined the actual effect of smartphone-based intervention alone on PA in children and adolescents which may provide additional information and be a valuable contribution to this area of inquiry.

### The Intervention of Two Smartphone Technologies and Their Effects

At present, the number of smartphone apps on the Chinese market monitored is 4.49 million, and youth per capita under 10, 10-14, and 15-19 years is as high as 30, 44, and 59, respectively [45]. These show that app technologies are mature enough to provide technical guarantees for the development of different interventions. Indeed, subgroup analysis found that app intervention can significantly improve PA. This finding is similar to previous meta-analyses on the adult population [14,21]. The advantage of app lies in its convenience and novelty. Through the app, you can receive feedback in real time, communicate, and self-monitor, among other possibilities. At present, an increasing number of children and adolescents are searching for health-related information and guiding their fitness via app [46,47]. Therefore, an app-based intervention meets the needs of modern people for health.

Unlike the intervention effect of an app, SMS text messaging intervention has no significant improvement effect on PA. However, 2 systematic reviews are inconsistent with the results of this review. Ludwig et al [48] performed a systematic review of the efficacy of the intervention that uses SMS text messaging to improve PA and found that some studies have potential effects on improving PA in adolescents. Similarly, Feter et al [21] found that SMS text messaging intervention can significantly improve PA in adults. However, interventions in some studies included in these 2 reviews are SMS text messaging plus other components, so it is difficult to discern whether the actual effect comes from SMS text messaging or other interventions. Unfortunately, there are no controlled trials on separate interventions for SMS text messaging–only and SMS text messaging plus other components, which is also an issue that researchers need to study further.

### Effects of the Smartphone on Different Age and Study Duration

Our subgroup analyses found that smartphone intervention has a significant effect on improving PA of children. In the studies in this review where the participants are children, the implementation of interventions requires parental assistance. A previous study found that parents play an important role in supporting and managing child-related health behaviors (eg, PA, sedentary behavior) [49]. The assistance of parents is conducive to the implementation of the intervention, which may lead to a positive effect on increasing PA. For adolescents, smartphone intervention has played a role in the intervention to a certain extent. However, adolescence is a transition period from the growth of children to adults, and it is also a stage of emotional fluctuation and frequent physiological changes. Rebellious emotions in the adolescent stage may resist and not cooperate with the implementer, which affects the effectiveness of the intervention and the compliance with the research.

The short-term (≤8 weeks) intervention effects may be attributed to the curiosity of the participants in the early stages of the intervention, and that they are willing to participate in the implementation. Over time, the decline in the interest and compliance of the participants led to the intervention effect not being maintained. A 4-week game app intervention found that the first-week intervention significantly improved PA in children, but the second-week and the fourth-week follow-up had no significant effect [22]. When all the games are unlocked or participants are familiar with the game, the participants are no longer interested in continuing, and the intervention effect of PA cannot be maintained. Therefore, considering the interest and passion of children and adolescents, we should strive to propose a novel strategy along with the design for a long-duration intervention.

### Strengths and Limitations

This review has several strengths. First, scientifically rigorous RCT studies were included in the meta-analysis. Second, the included studies are smartphone-alone intervention, excluding studies with other intervention content, so the results can better reflect the intervention effect of smartphone. Lastly, this review conducted a subgroup analysis to explore the potential modifying effect of different factors thoroughly.

Despite these strengths, the review has several limitations. First, there are not enough studies to examine potential modifying effects of LPA, economic levels, and demographic characteristics (eg, gender, body mass index, economic status). Second, the different characteristics of the included studies lead to high heterogeneity. However, we have included the latest Chinese and English literature and conducted a subgroup analysis based on literature characteristics.

### Conclusions

The findings of this meta-analysis indicated that interventions based on smartphone may be a promising strategy to increase the number of steps and TPA of children and adolescents, but the effect of intervention on MVPA remains to be studied. Currently, app intervention may be a more effective strategy among smartphone intervention technologies. To extend the promise of smartphone intervention, the future needs to design comparative trials among different smartphone technologies (ie, app vs SMS text messaging, app vs app + SMS text messaging, SMS text messaging vs app + SMS text messaging). Moreover, additional studies are needed to determine the effects on different participants, such as for children who are overweight and obese and low-income people.

## Appendix

Multimedia Appendix 1 Search strategy.

Multimedia Appendix 2 Characteristics of Included Studies.

Multimedia Appendix 3 Robustness of the Results.

## Abbreviations

LPA: light-intensity physical activity
mHealth: mobile health
MVPA: moderate-to-vigorous-intensity physical activity
PA: physical activity
PRISMA: preferred reporting items for systematic reviews and meta-analyses
RCT: randomized controlled trial
SMD: standardized mean difference
TPA: total physical activity
WHO: World Health Organization
WMD: weighted mean difference
