# Supplementary material for: Effects of Smartphone-Based Interventions on Physical Activity in Children and Adolescents: Systematic Review and Meta-analysis
Source: JMIR Mhealth Uhealth. 2021 Feb 1;9(2):e22601. doi: 10.2196/22601 (PMC7884215; doi:10.2196/22601)
Supplement: Multimedia Appendix 1 [file mhealth_v9i2e22601_app1.docx]

Multimedia Appendix 1. Search strategy

Taking PubMed as an example

(child*[tiab] OR pediatric[tiab] OR elementary[tiab] OR pupil[tiab] OR “primary [school](C:/Users/admin/Downloads/%E6%9C%89%E9%81%93/Dict/8.5.1.0/resultui/html/index.html#/javascript:;)”[tiab] OR “middle school”[tiab] OR “secondary school”[tiab] OR “high school”[tiab] OR [adolescen*](http://www.baidu.com/link?url=K6VUpVOA94EDBroPYY5UbhkneqUqdle6DvMd6blWk02ha8Dhh4pjuBkVjlJAGdD59nqpASFmipJBVIx5u3Bj5XPqIJ1fa9ybAb1Dv19aUJy)[tiab] OR teen*[tiab])

AND

(smartphone[tiab] OR “smart-phone”[tiab] OR cellphone[tiab] OR “cellular phone”[tiab] OR “mobile phone”[tiab] OR “mobile technolog*”[tiab] OR tablet[tiab] OR Actigraphy[tiab] OR Accelorometer[tiab] OR “Activity Tracker*”[tiab] OR pedometer*[tiab] OR “mobile application*” [tiab] OR APP OR “Mobile Exergame” OR “mobile game” OR “Text messag*” OR “Text Messaging Service”[tiab] OR “short message service”[tiab] OR SMS[tiab] OR “social media”[tiab] OR Facebook[tiab] OR mhealth[tiab] )

AND

(“physical activity”[tiab] OR PA [tiab] OR activit*[tiab] OR exercis*[tiab] OR steps[tiab] OR sport[tiab] OR inactivit*[tiab] OR “health behavio*”[tiab] )

AND

(“randomized controlled trial”[pt] OR “controlled clinical trial”[pt] OR random*[tiab] OR trial[tiab] )
