# Supplementary material for: Effects of Smartphone-Based Interventions on Physical Activity in Children and Adolescents: Systematic Review and Meta-analysis
Source: JMIR Mhealth Uhealth. 2021 Feb 1;9(2):e22601. doi: 10.2196/22601 (PMC7884215; doi:10.2196/22601)
Supplement: Multimedia Appendix 2 [file mhealth_v9i2e22601_app2.docx]

Multimedia Appendix 2. Characteristics of Included Studies

| **Author, year** | **Country** | **Sample (m/f)** | **Age (mean±SD)** | **Duration** | **Intervention type** | **Description of** **intervention** | **Control** | **Measurement** | **Outcome** |
| --- | --- | --- | --- | --- | --- | --- | --- | --- | --- |
| Garde [22], 2018 | Canada | 42(19/23) | 10.6±0.51 | 2 weeks | APP^a^ | Mobile Exergame | Self-monitoring | Objective: TAM^b^ | TPA^c^; step |
| Chen [23], 2017 | USA | 40(23/17) | 15 | 6 months | APP+SMS^d^ | Monitoring+feedback +encouragement | Self-monitoring | Subjective | PA days^e^ |
| Mendoza[24], 2017 | USA | 59(24/35) | 16.6±1.5 | 10 weeks | APP+SMS | Monitoring+feedback +goal-setting+encouragement | usual | Objective: AM^f^ | MVPA^g^ |
| Garde[25], 2016 | Canada | 42(26/16) | 11.3 ±1.2 | 2 weeks | APP | Mobile Exergame | Self-monitoring | Objective: TAM | TPA; step |
| Direito[26], 2015 | New Zealand | 35(16/19) | 15.67±1.1 | 8 weeks | APP | immersive game app | usual | Objective: AM | MVPA |
|  |  | 34(14/20) |  |  |  | nonimmersive game app |  |  |  |
| Garde[27], 2015 | Canada | 47(16/31 ) | 10.2±1.2 | 2 weeks | APP | Mobile Exergame | Self-monitoring | Objective: TAM | TPA; step |
| Armstrong[28], 2017 | USA | 101(39/62) | 9.9±2.7 | 3 months | SMS | Strengthen goals+ encouragement | usual | Subjective | MVPA |
| Thompson[29], 2016 | USA | 80(35/45) | 14-17 | 12 weeks | SMS | Strengthen goals+action reminders | usual | Objective: AM; Pedometer | MVPA;  step |
| Newton[30], 2009 | New Zealand | 78 | 14.4±2.37 | 12 weeks | SMS | Goal setting+action reminders | usual | Subjective | MVPA |
|  |  |  |  |  |  |  |  | Pedometer | Step |

^a^APP: application.

^b^TAM: tractivity activity monitor.

^c^TPA: total physical activity.

^d^SMS: short-message service.

^e^PA days: physical activity days per week.

^f^AM: accelerometer.

^g^MVPA: moderate-vigorous intensity physical activity.
