# Supplementary material for: Effects of Smartphone-Based Interventions on Physical Activity in Children and Adolescents: Systematic Review and Meta-analysis
Source: JMIR Mhealth Uhealth. 2021 Feb 1;9(2):e22601. doi: 10.2196/22601 (PMC7884215; doi:10.2196/22601)
Supplement: Multimedia Appendix 3 [file mhealth_v9i2e22601_app3.pdf]

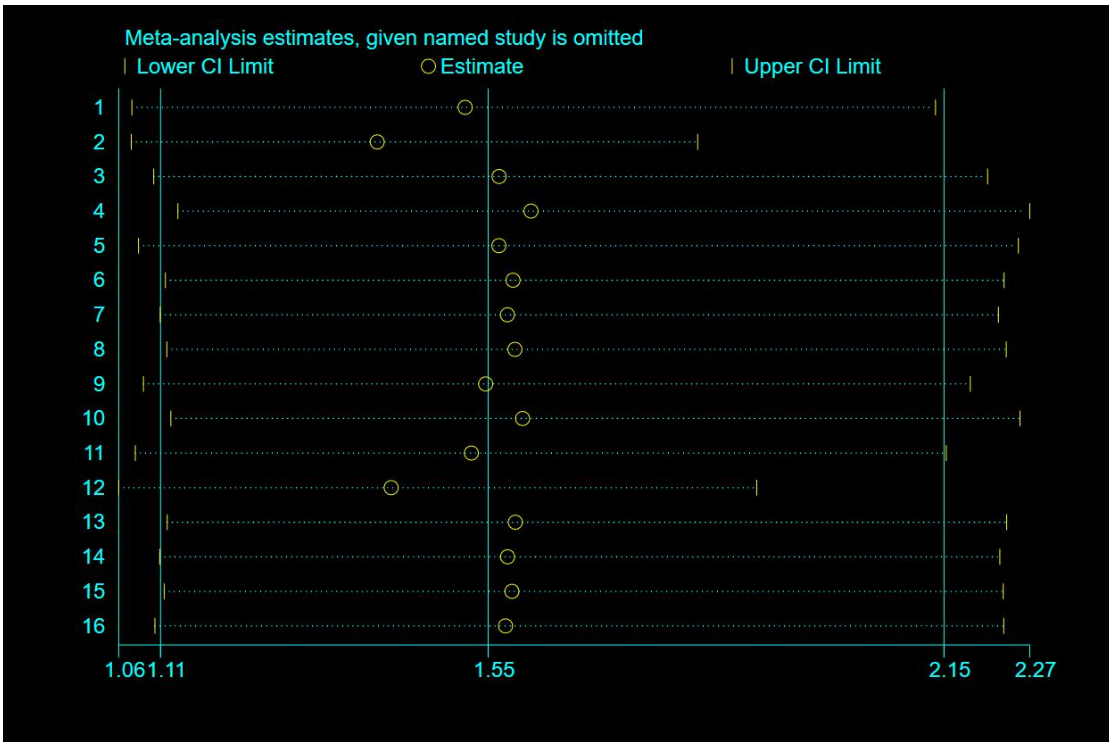

notes: 1, Garde (2018) - TPA; 2, Garde (2016) - TPA; 3, Garde (2015) - TPA; 4, Mendoza (2017) - MVPA; 5, Armstrong (2017) - MVPA; 6, Thompson(a) (2016) - MVPA; 7, Thompson(b) (2016) -MVPA; 8, Direito(a) (2015) - MVPA; 9, Direito(b) (2015) - MVPA; 10, Newton (2009) - step; 11, Garde (2018) - step; 12, Garde (2016) - step; 13,Thompson(a) (2016) - step; 14,Thompson(b) (2016) - step; 15,Garde(2015) - step; 16, Newton (2009) - step.

One study was removed each time to perform a meta-analysis again. The results of the effect did not change significantly, which indicates that the results of the meta-analysis in this study were reliable.
